# MiR-25-3p regulates the differentiation of intramuscular preadipocytes in goat via targeting *KLF4*

**DOI:** 10.5194/aab-64-17-2021

**Published:** 2021-01-18

**Authors:** Yu Du, Yue Zhao, Yong Wang, Qingyong Meng, Jiangjiang Zhu, Yaqiu Lin

**Affiliations:** 1 Key Laboratory of Qinghai-Tibetan Plateau Animal Genetic Resource Reservation and Utilization, Ministry of Education, Southwest Minzu University, Chengdu, China; 2 Qinghai-Tibetan Plateau Animal Genetic Resource Reservation and Utilization Key Laboratory of Sichuan Province, College of Animal Science and Veterinary Medicine, Chengdu, China; 3 Institute of Qinghai-Tibetan Plateau, Chengdu 610041, China; 4 State Key Laboratory of Agrobiotechnology, College of Biological Sciences, China Agricultural University, Beijing 100193, China

## Abstract

Adipocyte differentiation, which plays an important role in fat deposition, involves a complex molecular mechanism. MicroRNAs (miRNAs) are essential in this progress. Here, we showed that miR-25-3p expression had increased during goat intramuscular preadipocyte differentiation, which peaked at day 3. Using liposome transfection and qRT-PCR techniques, we found that knocking down miR-25-3p reduced the accumulation of lipid droplets by downregulating or upregulating the expression of *LPL*, *PPAR* γ, *AP2*, *SREBP1*, and *C/EBP* β but upregulating the expression of *KLF4*. Overexpression of miR-25-3p results in the opposite. Furthermore, the dual luciferase assay showed that overexpression of miR-25-3p significantly inhibited luciferase activity of *KLF4*. These results showed that miR-25-3p has a binding site within the 3${}^{\prime }$-UTR of *KLF4* mRNA. Together, these findings indicate that miR-25-3p is a positive regulator of intramuscular preadipocyte differentiation via targeting to *KLF4* in goats.

18 January 2021

## 1 Introduction

Intramuscular fat (IMF) is a type of fatty tissue deposited between skeletal muscle fibers and muscle bundles, which is regulated by the number and size of preadipocytes in the muscle, and it is a key factor affecting meat tenderness, flavor, and juiciness (Jiang et al., 2019; Ren, 2019). Studies have shown that multiple signaling pathways and their target genes can regulate fat deposition via regulating the process of adipocyte formation, proliferation, differentiation, and maturation (Grieco et al., 2019; Hafner et al., 2010; Son et al., 2014).

MicroRNAs are a class of endogenous single-stranded non-coding small RNAs about 18–25 nucleotides long. Studies have shown that microRNAs (miRNAs) can participate in a variety of biology pathway and cell functions, because the seed sequence of miRNAs can combine with the 3${}^{\prime }$ untranslated region (3${}^{\prime }$-UTR) of the target gene and regulate the expression of target gene by the way of inhibiting protein translation or degrading its mRNA (Lin et al., 2019; Zhou et al., 2013). Numerous studies have demonstrated the important role of miRNAs in regulating adipogenesis and lipid metabolism (Engin, 2017; Irani and Hussain, 2015; Zaiou et al., 2018; Qi et al., 2019; Lin et al., 2019; Acharya et al., 2019). For example, a study found that the expression of miR-425 may be controlled by *PPAR* γ during the adipogenesis process of adipocytes. Overexpression of miR-425 inhibited the proliferation of 3T3-L1 precursor adipocytes but significantly accelerated cellular adipogenic differentiation (Qi et al., 2019). Furthermore, miR-130a promotes the differentiation of mouse bone marrow mesenchymal stem cells (BMSCs) by negatively regulating the expression of Smurf2 (Lin et al., 2019)

Knockout of all miR-26-encoding loci can cause adipose tissue to swell rapidly in normally fed adult mice. However, miR-26a transgenic overexpressing mice are protected from obesity induced by a high-fat diet. Interestingly, miR-26 can restrain the differentiation and adipogenesis of adipocyte progenitor cells (APC) by targeting *Fbxl19* (Acharya et al., 2019).

miR-25-3p is a member of the miR-106b∼25 cluster (Wu et al., 2019) and has been reported to be involved in proliferation (Feng et al., 2014), apoptosis (Zhang et al., 2012), and motility (Xiang et al., 2015). Furthermore, previous studies have shown that the miR-106b∼25 cluster could regulate atherosclerosis in mice. The study found that, at 36 weeks of age, the lesion size of MiR-106b∼25 and *ApoE* double-knockout mice was 2 times smaller than that of *ApoE* knockout mice (Semo et al., 2019). In addition, miR-25 can inhibit triacylglycerol synthesis and lipid accumulation in goat mammary epithelial cells by negatively regulating *PGC*-1beta expression levels (Ma et al., 2018). These studies suggest that miR-25-3p may play an important role in regulating lipid metabolism and adipocyte differentiation.

Here, we investigated the role of miR-25-3p in adipogenesis of goat intramuscular preadipocytes and explored the underlying mechanism by identifying the involved factors. Our results show that overexpression of miR-25-3p can promote the expression level of key adipogenic regulatory genes. Then we examined the regulatory relationship between miR-25-3p and its putative target gene *KLF4*, which was demonstrated by luciferase reporter assay.

## 2 Materials and methods

### 2.1 Experimental animals

The goat intramuscular preadipocytes was obtained from the two male Jian Zhou goats (Sichuan, China) whose age was 5–7 days old. All experimental procedures were reviewed and approved by the Institutional Animal Care and Use Committee, Southwest Minzu University (Chengdu, Sichuan, China), and all the experiments complied with the requirements of the directory of the Ethical Treatment of Experimental Animals of China.

### 2.2 Cell culture and adipogenic differentiation

Detailed procedure for the collection and culture of intramuscular preadipocytes have previously been published (Xu et al., 2018). The F1 of goat intramuscular preadipocytes was cultured in DMEM/F12 (Hyclone, USA), containing 10 % (v/v) fetal bovine serum (FBS, Hyclone, USA), and put in a humidified incubator at 5 % CO${}_{2}$ and 37 ${}^{\circ }$C. The F3 cells were seeded into 12-well plates, and then the goat intramuscular preadipocytes reached 80 % confluence and were adipogenically induced by DMEM/F12 containing 10 % FBS and 50 µmol L${}^{-1}$ oleic acid (Shang et al., 2014). The medium was changed every two days until day 6.

### 2.3 Cell transfection

Before transfection, miR-25-3P mimics, inhibitor and NC were put into centrifuge at 2000 rpm for 10 min, then added 250  µL RNase Free H${}_{2}O$ to dissolve to 20 µM, stored at -20 ${}^{\circ }$C. When the preadipocytes reached 80 % of the plates, the goat intramuscular preadipocytes were transfected with Opti-MEM (Gibco-BRL Co. LTD), negative control (NC) (GenePharma, Shanghai, China), miR-25-3P mimics, and miR-25-3P inhibitor using Lipofectamine 3000 (Invitrogen, Carlsbad, USA) according to the manufacturer's instructions. After 6 h, the original medium was replaced by fresh differentiation medium to induce preadipocyte differentiation, and cells were collected after 48 h for RNA extraction.

### 2.4 RNA extraction and qRT-PCR

Total RNA of cells was extracted using TRIzol (TaKaRa, Otsu, Japan) and stored at -80 ${}^{\circ }$C. Reverse transcription of mRNA was performed using the Revert Aid First Strand cDNA Synthesis Kit (TaKaRa, Otsu, Japan) according to the manufacturer's instructions. Reverse transcription reactions for miRNA were performed using Mir-X™ miRNAs First-Strand Synthesis Kit (TaKaRa, Otsu, Japan) as per the manufacturer's instructions. U6 small nucleolar RNA and ubiquitously expressed transcript (UXT) were used as an endogenous control for miRNA and mRNA, respectively. The sequences of primers used are listed in Table 1. Abundance of mRNA for each gene was measured using LineGene 9600 Plus real-time PCR detection system (Bioer, Hangzhou China). The reaction volume for real-time PCR was 20 µL and consisted of 1 µL cDNA, 1 µL reverse and forward primers (per gene), 7 µL double-distilled water, and 10 µL TB Green™ Premix Ex Taq™ II (Takara, Otsu, Japan). The sequences of primers used are listed in Table 2. Relative gene expression levels were determined by the 2${}^{-\Delta \Delta \mathrm{Ct}}$ method (Rao et al., 2013).

**Table 1:** RNA oligonucleotides in this article.

Name	Sequence (5${}^{\prime }$–3${}^{\prime }$)
miR-25-3p mimic	CAUUGCACUUGUCUCGGUCUGA
	AGACCGAGACAAGUGCAAUGUU
Negative mimic	UUCUCCGAACGUGUCACGUTT
	ACGUGACACGUUCGGAGAATT
miR-25-3p inhibitor	UCAGACCGAGACAAGUGCAAUG
Negative inhibitor	CAGUACUUUUGUGUAGUACAA

**Table 2:** Primers utilized in this study.

Gene	Reference in GenBank	Primer sequence (5${}^{\prime }$–3${}^{\prime }$)	Tm (${}^{\circ }$C)	Product size (bp)
*PPAR*γ	NM_001285658.1	F: AAGCGTCAGGGTTCCACTATG	60	197
		R: GAACCTGATGGCGTTATGAGAC		
*AP2*	NM_001285623.1	F: TGAAGTCACTCCAGATGACAGG	58	143
		R: TGACACATTCCAGCACCAGC		
*LPL*	NM_001285607.1	F: TCCTGGAGTGACGGAATCTGT	60	174
		R: GACAGCCAGTCCACCACGAT		
*C/EBP*β	XM_018058020.1	F: CAAGAAGACGGTGGACAAGC	66	204
		R: AACAAGTTCCGCAGGGTG		
*SREBP1*	NM_001285755.1	F: AAGTGGTGGGCCTCTCTGA	58	127
		R: GCAGGGGTTTCTCGGACT		
*UXT*	XP_005700899.1	F: GCAAGTGGATTTGGGCTGTAAC	60	180
		R: TGGAGTCCTTGGTGAGGTTGT		
*U6*	NR_138085.1	F: GGAACGATACAGAGAAGATTAGC	64	189
		R: TGGAACGCTTCACGAATTTGCG		
*KLF4*	KU041754.1	F: GTCGGTCATCAGTGTTAGCAAAGG	62	126
		R: ACGGTGCACGAGGAGACAGTCT		
miR-25-3p	MIMAT0036100	CATTGCACTTGTCTCGGTCTGA	61	–

Note: F – sense primer; R – antisense primer.

### 2.5 Oil red O staining

As described in a previous investigation with minor modifications (Xu et al., 2019), the cells were cultured at 24-well plates and visualized by oil red O staining. The differentiated adipocytes were fixed with 10 % formaldehyde for 30 min and washed twice with PBS. The fixed adipocytes were staining with oil red O working solution for 20 min. After that, the cells were washed three times with PBS and photographed under a microscope.

### 2.6 Luciferase reporter assay

The 3${}^{\prime }$-UTR of *KLF4*, containing the miR-25-3p targeted site, was cloned using primers with NheI and XbaI (Thermo, MA, USA) cleavage sites. The wild-type 3${}^{\prime }$-UTR fragment was inserted into the corresponding site of the pmirGLO dual-luciferase vector (Promega, Madison, WI, USA). Then we co-transfected into 293T cells with miR-25-3p mimics, mimic NC, pmirGLO, and pmirGLO-*KLF4*. Cells were harvested after transfection 48 h, and then the dual-luciferase activity was analyzed using the Dual-Luciferase Reporter Assay System kit (Promega, Madison, WI, USA) according to the manufacturer's instructions.

### 2.7 Statistical analysis

Data are expressed as the “Means ± SEM” of three replicates in each experiment and analyzed using GraphPad Prism 5.0 (GraphPad Inc. USA) software. A t test was performed by SPSS 18 software (SPSS Inc. Chicago, IL, USA) to determine statistical differences between two groups. Comparisons between multiple groups were carried out using one-way analysis of variance. P<0.05 was considered statistically significant.

## 3 Results

### 3.1 Expression pattern of miR-25-3p during differentiation of goat intramuscular preadipocytes

**Figure 1:**
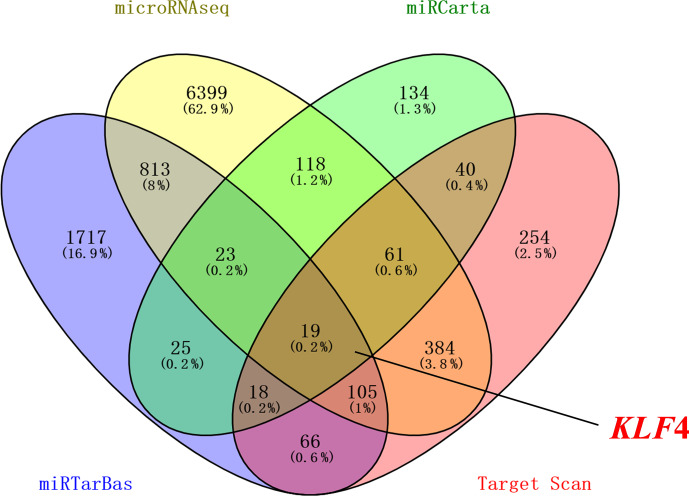
The target genes of miR-25-3p. Using Target Scan, miRCarta, miRTarBas, and microRNAseq found *KLF4* as a target gene of miR-25-3p.

**Figure 2:**
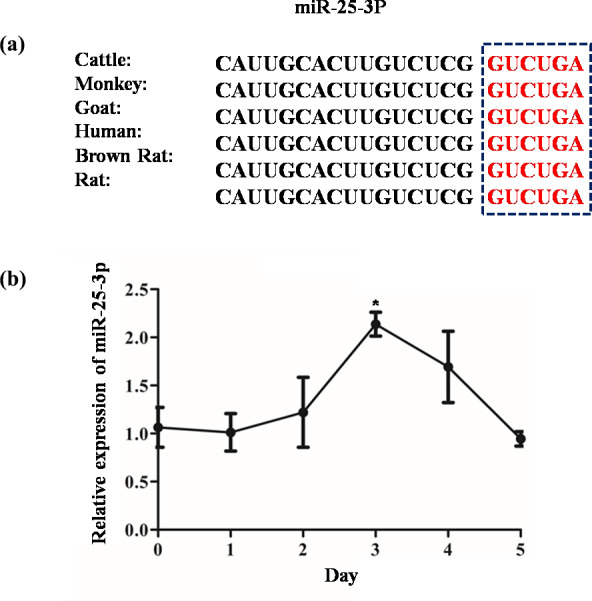
The relative expression levels of miR-25-3p during differentiation of goat intramuscular preadipocytes. **(a)** Mature miR-25-3p seed region was highly conserved among mammals. **(b)** miR-25-3p expression pattern during differentiation of goat preadipocytes.

Four databases were used to predict the possible target genes of miR-25-3p: Target Scan (http://www.targetscan.org/vert_71/, last access: 6 October 2020), miRCarta (https://mircarta.cs.uni-saarland.de, last access: 6 October 2020), miRTarBas (http://mirtarbase.mbc.nctu.edu.tw/index.html, last access: 6 October 2020), and microRNAseq (https://www.encodeproject.org/microrna/microrna-seq/, last access: 6 October 2020). We found 19 common target genes using Venny 2.1 online software. Then we selected *KLF4* related to fat as the target gene (Fig. 1). The seed region of miR-25-3p is highly conserved among mammals (Fig. 2a). To investigate the regulation of miR-25-3p on adipocyte deposition, qRT-PCR was used to detect the expression pattern of miR-25-3p from day 0 to day 5 before adipocyte differentiation. As shown in Fig. 2b, miR-25-3p expression gradually increased during day 0 to day 3, peaked on day 3, and decreased after that. These results indicated that miR-25-3p may play an important role during goat intramuscular adipocyte differentiation.

### 3.2 Knockdown miR-25-3p inhibits goat preadipocyte differentiation

**Figure 3:**
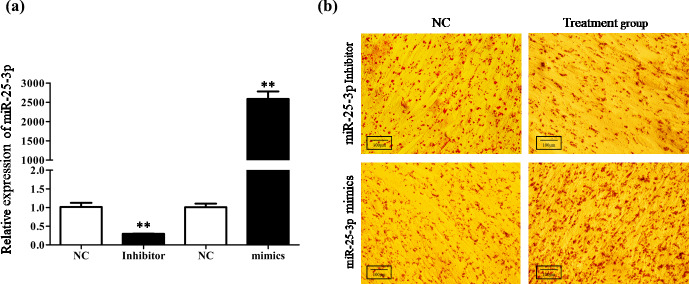
Knockdown miR-25-3p decreased the formation of lipid droplets in goat preadipocyte differentiation. **(a)** The transfection efficiency of miR-25-3p mimics and miR-25-3p inhibitor was detected by qRT-PCR, compared with the negative control. **(b)** After 48 h of preadipocyte differentiation, cells were fixed and stained with Oil Red O.

**Figure 4:**
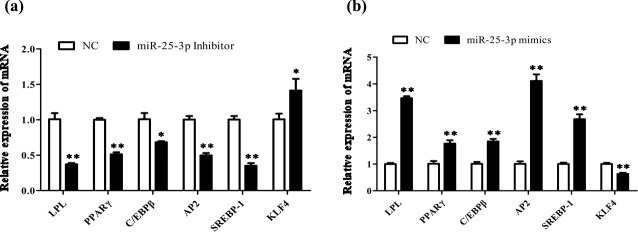
Overexpression of miR-25-3p promotes goat intramuscular differentiation. **(a)** The mRNA expression levels of key adipogenic regulatory genes were detected after transfected miR-25-3p inhibitor in goat intramuscular preadipocytes. **(b)** The mRNA expression levels of key adipogenic regulatory genes were detected after transfected miR-25-3p mimics in goat intramuscular preadipocytes.

To investigate the role of miR-25-3p in cell differentiation, goat intramuscular preadipocytes were transfected with miR-25-3p mimics or miR-25-3p inhibitor during the differentiation period. The qRT-PCR technique was used to determine the transfection efficiency of miR-25-3p after 48 h. The results showed that the expression levels of miR-25-3p were significantly increased upon miR-25-3p mimic transfection and decrease in response to miR-25-3p inhibitor transduction compared with the NC group (P<0.01; Fig. 3a). The oil red staining showed that knocking down the expression of miR-25-3p significantly decreased the formation of lipid droplets (Fig. 3b). In contrast, overexpression of miR-25-3p visibly increased lipid accumulation. These results suggested that miR-25-3p can promote goat intramuscular adipogenesis. To further investigate the potential molecular mechanism of miR-25-3p in goat intramuscular preadipocytes, we detected the expression of key adipogenic regulatory genes in adipocytes. Compared with the negative control, after knocking down miR-25-3p in goat intramuscular preadipocytes, the mRNA expression levels of lipoprotein lipase (*LPL*), peroxisome proliferator-activated receptor gamma (*PPAR* γ), adipocyte fatty acid-binding protein (*AP2*), sterol-regulatory element-binding proteins (*SREBP1*), and CCAAT/enhancer-binding protein (*C/EBP* β) were significantly reduced. However, the expression of the target gene *KLF4* was significantly increased (Fig. 4a). As expected, overexpression of miR-25-3p showed the opposite result (Fig. 4b). These results indicated that miR-25-3p may play a positive regulatory role during the differentiation of goat intramuscular preadipocytes.

### 3.3 *KLF4* as a target gene of miR-25-3p

**Figure 5:**
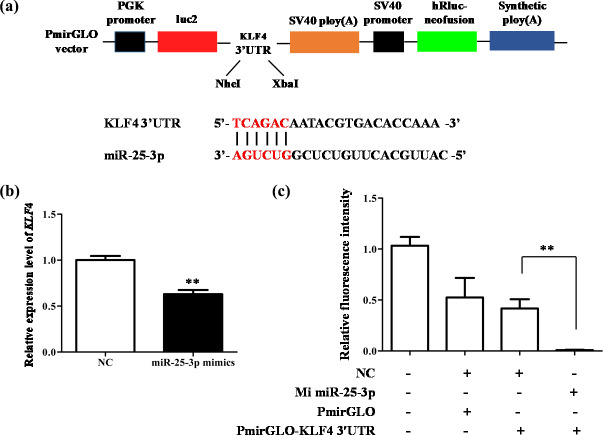
miR-25-3p can target 3${}^{\prime }$-UTR of *KLF4*. **(a)** Schematic diagram of pmirGLO dual luciferase and the interaction between miR-25-3p and *KLF4*. **(b)** The mRNA expression level of *KLF4* after miR-25-3p overexpression. **(c)** *KLF4* 3${}^{\prime }$-UTR dual luciferase vector was co-transfected with miR-25-3p mimics and negative control.

The above research proves that miR-25-3p plays an important role in the differentiation of adipocytes. To further explore its molecular mechanism, we used luciferase reporter assay to verify the relationship between *KLF4* and miR-25-3p (Fig. 5a). The result showed that when miR-25-3p was overexpressed, the mRNA expression of *KLF4* was significantly inhibited (Fig. 5b). As expected, miR-25-3p mimics and *KLF4* 3${}^{\prime }$-UTR dual-luciferase reporter vectors co-transfected into 293T cells significantly reduced the activity of the pmirGLO–*KLF4*–3${}^{\prime }$-UTR reporter gene, suggesting that miR-25-3p can target the 3${}^{\prime }$-UTR of *KLF4* (Fig. 5c).

## 4 Discussion

The important role of fat is to store energy and maintain metabolic stability. According to the location of fat deposition, it can be divided into four categories: subcutaneous fat, intermuscular fat, intramuscular fat, and visceral fat. Among them, intramuscular fat deposition is of great significance to meat flavor, nutrition, and taste (Cui et al., 2016; Poleti et al., 2018a; Wood et al., 2008). In addition, intramuscular fat cells can gradually accumulate through the proliferation and differentiation to form the marble pattern in meat (Poleti et al., 2018b; Zhao et al., 2019). In recent years, miRNAs have become a hot spot for studying the processes of fat differentiation and lipid metabolism. For example, miR-142 and miR-144 can target *FoxO1* and inhibit its expression level in sheep precursor adipocytes while promoting the differentiation of adipocytes via the negative regulation of *PPAR* γ by *FoxO1* (Zhao, 2018). In addition, miR-331-3p can inhibit the proliferation of porcine precursor adipocytes. Overexpression of miR-331-3p can regulate the expression of dihydrolipamide S-succinyltransferase (DLST) to regulate fatty acid accumulation in the citrate pyruvate cycle (Chen et al., 2019). However, the molecular mechanism underlying the role of miR-25-3p in lipogenesis is not fully understood.

In this experiment, we investigated the role of miR-25-3p in regulating goat intramuscular preadipocyte differentiation. We found that the expression of miR-25-3p was gradually increased in the early phase and then decreased. We also showed that overexpression of miR-25-3p promoted, whereas downregulation of miR-25-3p suppressed goat intramuscular preadipocyte differentiation and lipid accumulation. This suggests that miR-25-3p plays a positive role in intramuscular preadipocyte differentiation in goats. Previous research has shown that miR-25 suppressed 3T3-L1 adipogenesis by targeting *KLF4* and *C/EBP* α (Liang et al., 2015). Moreover, the latest research demonstrated that exosomal miR-25-3p could promotes vascular permeability and angiogenesis in mouse vascular endothelial cells by targeting and inhibiting the expression level of *KLF4* (Zeng et al., 2018). We think that this could be a difference between from different species or organs. Noteworthy is the fact that the expression of key adipogenic genes in our study also had dramatic changes.

*LPL* is a rate-limiting enzyme that can be widely distributed in adipose tissue. In brown adipose tissue, *LPL* is related to thermogenesis. In white adipose tissue, the increase in *LPL* activity can help lipid storage (Nimonkar et al., 2020; Pérez-Torres et al., 2019; Ruppert and Kersten, 2020). *PPAR* γ is a member of the nuclear receptor family and a necessary transcription factor for adipogenesis. The functions of *PPAR* γ are to promote the expression of genes involved in adipogenesis and maintaining mature adipocytes (Jeon et al., 2020). Furthermore, in goat mammary epithelial cells *PPAR* γ could significantly up-regulate the expression of genes that are related to synthesis and secretion of promoting fat deposition, such as *LPL*, *FASN*, *ACACA*, *PLIN3*, *FABP3*, *PLIN2*, and *SREBF*1 (Shi et al., 2013). *AP2* is a fatty acid binding protein that promotes the hydrolysis and transport of lipids in the cytoplasm (Zhang et al., 2019). Studies have shown that overexpression of *KLF9* in chicken precursor adipocytes can inhibit the accumulation of triglycerides by down-regulating *AP2* (Sun et al., 2019). *SREBP1* can regulate the expression of genes related to fatty acid synthesis. Knockout *SREBP1* in human liver cancer cells can inhibit the synthesis of triglycerides and prevent the expression levels of fatty acid synthesis genes, such as *ACLY*, *ACACA*, and *FASN* (Edwards et al., 2000; Moon et al., 2001; Yang et al., 2019). In our study, overexpression of miR-25-3p significantly promoted the mRNA expression levels of *LPL*, *PPAR* γ, *AP2*, *SREBP1*, and *C/EBP* β. Together, these results further suggest that the fat-promoting effect of miR-25-3p can be attributed to inhibition of the key adipogenic genes during goat intramuscular preadipocyte adipogenesis.

To screen the targets of miR-25-3p and the possible binding sites between miR-25-3p and its target, using Target Scan, miRCarta, miRTarBas, and microRNAseq target prediction packages, we found that *KLF4* 3${}^{\prime }$-UTR can efficiently bind to the miR-25-3p seed region, which is highly conserved among mammals. *KLF4* is a member of *KLF*s, and a growing number of studies have confirmed the role of *KLF*s in regulating adipocyte differentiation (Lee et al., 2016; Ma et al., 2020). As expected, the luciferase assay showed that overexpression of miR-25-3p significantly reduced luciferase activity of *KLF4*, which was consistent with the mechanism of miRNAs targeting mRNA in goat intramuscular adipocytes. Previous studies have shown that overexpression of *KLF4* can inhibit the accumulation of lipid droplets in goat intramuscular preadipocytes (Lin, 2018). In addition, in mouse stromal vascular cells and both subcutaneous and visceral human fat, an adenosine receptor-Krüppel-like factor 4 protein axis inhibits adipogenesis (Anna et al., 2014). The qRT-PCR result in our study showed that overexpression of miR-25-3p inhibited the expression of *KLF4* exceedingly.

Taken together, these results further suggest that miR-25-3p plays a positive regulation in goat intramuscular preadipocyte via targeting *KLF4* and inhibiting its expression.

## 5 Conclusions

We demonstrated that miR-25-3p positively regulates the differentiation of goat intramuscular preadipocytes. Furthermore, we found that miR-25-3p promotes preadipocyte differentiation by regulating the expression of key adipogenic genes and binding to the 3${}^{\prime }$-UTR of *KLF4* and thereby inhibiting *KLF4* transcription.

## Data Availability

The original data are available upon request to the corresponding author.

## References

[bib1.bib1] Acharya A, Berry DC, Zhang H, Jiang Y, Jones BT, Hammer RE, Graff JM, Mendell JT (2019). MiR-26 suppresses adipocyte progenitor differentiation and fat production by targeting Fbxl19. Gene Dev.

[bib1.bib2] Anna E, Shannon HC, Hillary JC, Melissa F, Noyan G, Katya R (2014). An adenosine receptor-Krüppel-like factor 4 protein axis inhibits adipogenesis. J Biol Chem.

[bib1.bib3] Chen T, Cui J, Ma L, Zeng Y, Chen W (2019). The Effect of MicroRNA-331-3p on Preadipocytes Proliferation and Differentiation and Fatty Acid Accumulation in Laiwu Pigs. Biomed Res Int.

[bib1.bib4] Cui J, Chen W, Liu J, Xu T, Zeng Y (2016). Study on quantitative expression of PPARγ and ADRP in muscle and its association with intramuscular fat deposition of pig. SpringerPlus.

[bib1.bib5] Edwards PA, Tabor D, Kast HR, Venkateswaran A (2000). Regulation of gene expression by SREBP and SCAP. Biochim Biophys Acta.

[bib1.bib6] Engin AB (2017). MicroRNA and Adipogenesis. Adv Exp Med Biol.

[bib1.bib7] Feng SJ, Pan WJ, Jin Y, Zheng JH (2014). MiR-25 promotes ovarian cancer proliferation and motility by targeting LATS2. Tumour Biol.

[bib1.bib8] Grieco GE, Brusco N, Licata G, Nigi L, Formichi C, Dotta F, Sebastiani G (2019). Targeting microRNAs as a Therapeutic Strategy to Reduce Oxidative Stress in Diabetes. Int J Mol Sci.

[bib1.bib9] Hafner M, Landthaler M, Burger L, Khorshid M, Hausser J, Berninger P, Rothballer A, Ascano M, Jungkamp A-C, Munschauer M, Ulrich A, Wardle GS, Dewell S, Zavolan M, Tuschl T (2010). Transcriptome-wide identification of RNA-binding protein and microRNA target
sites by PAR-CLIP. Cell.

[bib1.bib10] Irani S, Hussain MM (2015). Role of microRNA-30c in lipid metabolism, adipogenesis, cardiac remodeling and cancer. Curr Opin Lipidol.

[bib1.bib11] Jeon YG, Lee JH, Ji Y, Sohn JH, Lee D, Kim DW, Yoon SG, Shin KC, Park J, Seong JK, Cho J-Y, Choe SS, Kim JB (2020). RNF20 Functions as a Transcriptional Coactivator for PPARγ by Promoting NCoR1 Degradation in Adipocytes. Diabetes.

[bib1.bib12] Jiang Q, Sun B, Liu Q, Cai M, Wu R, Wang F, Yao Y, Wang Y, Wang X (2019). MTCH2 promotes adipogenesis in intramuscular preadipocytes via an m6A-YTHDF1-dependent mechanism. FASEB J.

[bib1.bib13] Lee DS, Choi H, Han BS, Kim WK, Lee SC, Oh K-J, Bae K-H (2016). C-Jun regulates adipocyte differentiation via the KLF15-mediated mode. Biochem Biophys Res Commun.

[bib1.bib14] Liang WC, Wang Y, Liang PP, Pan XQ, Fu WM, Yeung VSY, Lu YF, Wan DCC, Tsui SKW, Tsang SY, Ma WB, Zhang JF, Waye MMY (2015). MiR-25 suppresses 3T3-L1 adipogenesis by directly targeting KLF4 and C/EBPα. J Cell Biochem.

[bib1.bib15] Lin S (2018). Regulation of KLF4 on intramuscular preadipocyte differentiation in goat.

[bib1.bib16] Lin Z, He H, Wang M, Liang J (2019). MicroRNA-130a controls bone marrow mesenchymal stem cell differentiation towards the osteoblastic and adipogenic fate. Cell Prolif.

[bib1.bib17] Ma C, Xia R, Yang S, Liu L, Zhang J, Feng K, Shang Y, Qu J, Li L, Chen N, Xu S, Zhang W, Mao J, Han J, Chen Y, Yang X, Duan Y, Fan G (2020). Formononetin attenuates atherosclerosis via regulating interaction between KLF4 and SRA in apoE-/-mice. Theranostics.

[bib1.bib18] Ma L, Qiu H, Chen Z, Li L, Zeng Y, Luo J, Gou D (2018). MiR-25 modulates triacylglycerol and lipid accumulation in goat mammary epithelial cells by repressing PGC-1beta. J Anim Sci Biotechnol.

[bib1.bib19] Moon YA, Shah NA, Mohapatra S, Warrington JA, Horton JD (2001). Identification of a mammalian long chain fatty acyl elongase regulated by sterol regulatory element-binding proteins. J Biol Chem.

[bib1.bib20] Nimonkar AV, Weldon S, Godbout K, Panza D, Hanrahan S, Cubbon R, Xu F, Trauger JW, Gao J, Voznesensky A (2020). A lipoprotein lipase-GPI-anchored high-density lipoprotein-binding protein 1 fusion lowers triglycerides in mice: Implications for managing familial chylomicronemia syndrome. J Biol Chem.

[bib1.bib21] Pérez-Torres I, Gutiérrez-Alvarez Y, Guarner-Lans V, Díaz-Díaz E, Manzano Pech L, Caballero-Chacón SDC (2019). Intra-Abdominal Fat Adipocyte Hypertrophy through a Progressive Alteration of Lipolysis and Lipogenesis in Metabolic Syndrome Rats. Nutrients.

[bib1.bib22] Poleti MD, Regitano LCA, Souza GHMF, Cesar ASM, Simas RC, Silva-Vignato B, Oliveira GB, Andrade SCS, Cameron LC, Coutinho LL (2018). Longissimus dorsi muscle label-free quantitative proteomic reveals biological mechanisms associated with intramuscular fat deposition. J Proteomics.

[bib1.bib23] Poleti MD, Regitano LCA, Souza GHMF, Cesar ASM, Simas RC, Silva-Vignato B, Oliveira GB, Andrade SCS, Cameron LC, Coutinho LL (2018). Data from proteomic analysis of bovine Longissimus dorsi muscle associated with intramuscular fat content. Data Brief.

[bib1.bib24] Qi R, Wang J, Wang Q, Qiu X, Yang F, Liu Z, Huang J (2019). MicroRNA-425 controls lipogenesis and lipolysis in adipocytes. BBA-Mol Cell Biol L.

[bib1.bib25] Rao X, Huang X, Zhou Z, Lin X (2013). An improvement of the $2^{-\Delta \Delta \mathrm{CT}}$ method for quantitative real-time polymerase chain reaction data analysis. Biostat Bioinforma Biomath.

[bib1.bib26] Ren L (2019). Biological Function Studies of Candidate Genes S100A10 and Bta-miR-210 Related to Intramuscular Fat Deposition in Cattle.

[bib1.bib27] Ruppert PMM, Kersten S (2020). A lipase fusion feasts on fat. J Biol Chem.

[bib1.bib28] Semo J, Chernin G, Jonas M, Shimoni S, George J (2019). Deletion of the Mir-106b∼25 MicroRNA cluster attenuates atherosclerosis in Apolipoprotein E knockout mice. Lipids Health Dis.

[bib1.bib29] Shang ZC, Guo L, Wang N, Shi H, Wang YX, Li H (2014). Oleate promotes differentiation of chicken primary preadipocytes in vitro. Biosci Rep.

[bib1.bib30] Shi H, Luo J, Zhu J, Li J, Sun Y, Lin X, Zhang L, Yao D, Shi H (2013). PPAR γ Regulates Genes Involved in Triacylglycerol Synthesis and Secretion in Mammary Gland Epithelial Cells of Dairy Goats. PPAR Res.

[bib1.bib31] Son YH, Ka S, Kim AY, Kim JB (2014). Regulation of Adipocyte Differentiation via MicroRNAs. Endocrinol Metab (Seoul).

[bib1.bib32] Sun GR, Zhang M, Sun JW, Li F, Ma XF, Li WT, Han RL, Li ZJ, Jiang RR, Li GX, Yan FB, Kang XT (2019). Krüppel-like factor KLF9 inhibits chicken intramuscular preadipocyte differentiation. Brit Poultry Sci.

[bib1.bib33] Wood JD, Enser M, Fisher AV, Nute GR, Sheard PR, Richardson RI, Hughes SI, Whittington FM (2008). Fat deposition, fatty acid composition, meat quality: A review. Meat Sci.

[bib1.bib34] Wu T, Hu H, Zhang T, Jiang L, Li X, Liu S, Zheng C, Yan G, Chen W, Ning Y, Li Y, Lu Z (2019). MiR-25 Promotes Cell Proliferation,
Migration, and Invasion of Non-Small-Cell Lung Cancer by Targeting the
LATS2/YAP Signaling Pathway. Oxid Med Cell Longev.

[bib1.bib35] Xiang J, Hang JB, Che JM, Li HC (2015). MiR-25 is up-regulated in non-small cell lung cancer and promotes cell proliferation and motility by targeting FBXW7. Int J Clin Exp Pathol.

[bib1.bib36] Xu Q, Lin S, Li Q, Lin Y, Xiong Y, Zhu J, Wang Y (2019). Fibroblast growth factor 21 regulates lipid accumulation and adipogenesis in goat intramuscular adipocyte. Anim Biotechnol.

[bib1.bib37] Xu Q, Lin S, Wang Y, Zhu J, Lin Y (2018). Fibroblast growth factor 10 (FGF10) promotes the adipogenesis of intramuscular preadipocytes in goat. Mol Biol Rep.

[bib1.bib38] Yang N, Li C, Li H, Liu M, Cai X, Cao F, Feng Y, Li M, Wang X (2019). Emodin Induced SREBP1-Dependent and SREBP1-Independent Apoptosis in Hepatocellular Carcinoma Cells. Front Pharmacol.

[bib1.bib39] Zaiou M, El Amri H, Bakillah A (2018). The clinical potential of adipogenesis and obesity-related microRNAs. Nutr Metab Cardiovasc Dis.

[bib1.bib40] Zeng Z, Li Y, Pan Y, Lan X, Song F, Sun J, Zhou K, Liu X, Ren X, Wang F, Hu J, Zhu X, Yang W, Liao W, Li G, Ding Y, Liang L (2018). Cancer-derived exosomal miR-25-3p promotes pre-metastatic niche formation by inducing vascular permeability and angiogenesis. Nat Commun.

[bib1.bib41] Zhang HY, Zuo Z, Lu X, Wang HY, Zhu ZL (2012). MiR-25 regulates apoptosis by targeting Bim in human ovarian cancer. Oncol Rep.

[bib1.bib42] Zhang Y, Zhou L, Zhang Z, Xu Q, Han X, Zhao Y, Song X, Zhao T, Ye L (2019). Effects of di (2-ethylhexyl) phthalate and high-fat diet on lipid metabolism in rats by JAK2/STAT5. Environ Sci Pollut R Int.

[bib1.bib43] Zhao X, Chen S, Tan Z, Wang Y, Zhang F, Yang T, Liu Y, Ao H, Xing K, Wang C (2019). Transcriptome Analysis of Landrace Pig Subcutaneous Preadipocytes during Adipogenic Differentiation. Genes (Basel).

[bib1.bib44] Zhao Y (2018). Study on miR-142 and miR-144 Down-Regulation Contributions to Differentiation of Ovine Rreadipocytes by Targeting FoxO1 Gene.

[bib1.bib45] Zhou P, Xu W, Peng X, Luo Z, Xing Q, Chen X, Hou C, Liang W, Zhou J, Wu X, Songyang Z, Jiang S (2013). Large-scale screens of miRNA-mRNA interactions unveiled that the 3${}^{\prime }$UTR of a gene is targeted by multiple miRNAs. PloS One.

